# How climate change and insecurity pushed 5 million people to hunger in Chad, Africa

**DOI:** 10.1002/puh2.47

**Published:** 2022-12-20

**Authors:** Shuaibu Saidu Musa, Tchindebe Bouri Ela, Emery Manirambona, Deborah Oluwaseun Shomuyiwa, Usman Abubakar Haruna, Don Eliseo Lucero‐Prisno, Abdulrahman Muhammad

**Affiliations:** ^1^ Department of Nursing Science Ahmadu Bello University Zaria Nigeria; ^2^ Provincial Health Delegation of Wadi‐Fira Baltine Chad; ^3^ College of Medicine and Health Sciences University of Rwanda Kigali Rwanda; ^4^ Faculty of Pharmacy University of Lagos Lagos Nigeria; ^5^ Department of Biomedical Sciences Nazarbayev University School of Medicine Nursultan Kazakhstan; ^6^ Department of Global Health and Development London School of Hygiene and Tropical Medicine London UK; ^7^ Faculty of Management and Development Studies University of the Philippines Open University Los Baños Laguna Philippines; ^8^ Faculty of Public Health Mahidol University Bangkok Thailand; ^9^ Department of Nursing Science University of Maiduguri Maiduguri Nigeria

**Keywords:** Chad, climate change, food security, hunger, insecurity, Lake Chad Basin

## Abstract

Climate change and insecurity pose challenges to food security around the globe. Chad has experienced several climate changes and insecurity influences on its food security, where, approximately 5 million people were pushed into hunger in the country. Desertification, flooding, and depletion of freshwater resources have pushed the country into hunger due to their negative effect on agro‐pastoral production in Chad. Insecurity due to the Boko‐Haram insurgency, in particular, has impaired agriculture, which is the mainstay of the country's economy. The influx of refugees from Nigeria and Cameroon has also compounded the hunger in Chad, as the country hosts the largest number of refugees in the region. Leveraging collaboration for climate change and improving security should be a priority for Chad. Increased consideration and action in the region can facilitate focus on climate change action in the region. International and multisectoral collaboration can set the pace for revamping the present security framework. Raising climate change awareness among key stakeholders and building capacity at the national level can help mitigate the impact of climate change on food security in Chad.

## INTRODUCTION

Climate change and insecurity have impacted food security around the world. They have impacted food availability, accessibility as well as quality at the global, regional, and local levels. The impact is prominent on the African continent and particularly, in Sub‐Saharan Africa. The Republic of Chad and other countries of the Sahel and West Africa are facing a food and nutrition crisis of exceptional proportions due to numerous conflicts and the negative impact of climate change. In these countries, acute food insecurity has almost quadrupled between 2019 and 2022, with millions more at risk of slipping into a crisis [1].

Hunger has recently become a severe problem in the Chad Republic. With 42 % of its citizens living in poverty, Chad has the highest hunger rates in the world in 2022 [2]. Due to climate change and insecurity, it is predicted that in 2022, more than 5.3 million people, 50% were women, would be food insecure in Chad [3]. Insecurity, especially in the Lake Chad Basin (LCB) region, has impacted food security in Chad. Violence perpetrated by insurgents and counter‐insurgency groups has hindered agricultural production, livelihood access, markets, trade and humanitarian aid. This has resulted in the forced displacement of 2.6 million people and acute food insecurity for 5 million people, as of January 2020 [4]. Although the whole country feels the impact, the situation is worst in the Sahel provinces, including Lac, where 20%–25% of the population is threatened by food insecurity [3].

## DISCUSSION

The effects of climate change in Chad range from perpetual temperature increment to environmental degradation. Desertification, flooding, and the disappearance of reliable freshwater resources are manifestations of environmental degradation [5]. The temperature increment in Chad occurs at rates that are above the global average. The average temperature in Chad is projected to increase by an average of 1°C, particularly in the northern part of the Sahel region and the entire Saharan zone [6]. The temperature rise is projected to be 1.5 times higher than in any place in the world [7]. This rise in temperatures also resulted in wildfires that have been degrading arable lands and causing loss of water resources for agriculture.

The historic recession of Lake Chad and the depletion of freshwater resources within the LCB are consequences of desertification resulting from variable precipitation and ever‐increasing temperatures [5], overuse and unplanned irrigation [4]. Lake Chad has a substantial contribution to agriculture. However, it has gradually shrunk by nearly ninety percent of its original size since the 1960s, impacting food security and livelihoods for 50 million people residing in the LCB [8]. Though the water levels were partly replenished in 1999 and have since stabilised, the region surrounding the lake remains vulnerable to unpredictable periods of intense rainfall, regular droughts and frequent floods, making it extremely difficult to sustain livelihoods [4] This has affected provisions from farming, fishing, livestock production, and transhumance, forcing many farmers to subsistence farming practices for survival, while some abandoned farming and moved into Chad, further straining the fragile food system in the country. Drought and recurrent rainfall deficits in Chad have caused degradation of natural resources; decline in agricultural, pastoral, and fishery produce and the erosion of biodiversity [6] and hence, negatively impacting food security in the country.

The arid nature of Chad makes it susceptible to flooding because the soil structure is sandy and loose, which, therefore, cannot hold much quantity of water. The 2020 rainy season in the country was marked by heavy rainfall that resulted to serious flooding. Of the 23 provinces in Chad, 20 were flooded, affecting 388,000 people [7]. The flood ruined food stocks, washed away thousands of cattle and destroyed 11,380 hectares of sown fields which deprived farmers of that season's food produce most of which were intended for household consumption [6, 7] and, therefore exposed them to hunger.

Heat stress as a result of the rising temperatures in Chad also affects livestock's health, resulting in poor growth, fertility reduction and low milk production or, worse, mortality in severe conditions. This has resulted in a loss of livelihoods for many Chadians as they are mainly agro‐pastoralists, further exposing them to poverty and hunger due to hikes in food prices across the country. In the wave of the hike in food prices, surveys suggested that 20% of Chadian households experienced high food prices between 2020 and 2021, and with the influence, running into 2022 [8], This can further impair the food purchasing power of many Chadians and result in severe hunger across the country.

The impact of deteriorating security has garnered attention in Chad. Insecurity has adversely affected the reliable access to safe, affordable, and nutritious food in Chad. The violent Boko‐Haram insurgency which originated in Maiduguri, Borno State – Northeastern Nigeria has particularly disrupted many agricultural activities in the LCB area. Since 2014 and until recently, the group has unleashed violence across the LCB region, involving Nigeria, Niger, Chad, and Cameroon [9, 10] which made it difficult for the countries involved to have absolute control over the natural resources in the region. Displacements and military‐enforced movement restrictions have hampered agricultural livelihoods. People were either hindered from accessing their farmlands or lost farmlands that serve as a source of income. Most times, harvests are raided by terrorists.

Farming of tall cereals such as maize, sorghum, and millet was prohibited by the military for fear that such farms could provide hiding places for launching attacks by the Boko‐Haram terrorist groups. Threats of being kidnapped or bombed by landmines have also prevented farmers from fully engaging in agricultural activities [4]. Uses of fertilizers on farms for better crop yields have been restricted because fertilizers can be used for the fabrication of improvised explosive devices by terrorists.

The bed of Lake Chad allows for herds to access forage and water during a deficit or pastoral lean period; however, Boko‐Haram‐related conflict and security restrictions imposed by local authorities prevent access to the area [4]. The situation made livestock rearing almost impossible and forced the migration of herders. This migration caused conflicts between herders and farmers as crops were being destroyed by the livestock movements, exacerbating insecurity and adding more pressure on food scarcity in Chad. Fishing is also an important source of food and income in Chad and contributes significantly to the local economy in the LCB; however, in 2020 environmental variability, conflict, and displacements have contributed to a 60% decline in fish production [4].

Conflict‐afflicted refugees have also added to the ongoing food insecurity in Chad. Chad hosts one of the largest numbers of refugees in the Sahel, with over 1 million people forcefully displaced in the country [2]. In 2022, Chad experienced an inflow of refugees that fled conflict in Nigeria and Cameroon. This has also put additional pressure on the already scarce resources in the country. Chad's Lac region, is one of the poorest areas in the country, hosting thousands of Nigerian refugees and internally displaced people [11]. Consequently, it is estimated that 1.7 million people in Chad will be severely food insecure from June to September 2022, making it the third consecutive year of severe food insecurity and the worst lean season in the country, in the last decade [2].

## RECOMMENDATIONS

Leveraging regional collaborations could solve the challenges posed by climate change and insecurity in agriculture, particularly at the LCB. Chad and the other three countries of the LCB should join forces to fight the insurgency and improve security in the region. Collaborations on climate action should also be improved. These collaborations should encompass the collective conception and strategic adoption of innovative trends for environmental sustainability. Raising awareness of key stakeholders about climate change and its impact can help improve food security in Chad. Governments and non‐government leaders must collaborate to address the complex intricacies of climate change and insecurity.

Humanitarian actions are important in adapting to social and economic instability associated with climate change and insecurity. Food, shelter, water and sanitation provisions for the refugees in Chad should be enhanced by humanitarian organizations. Interventions, including humanitarian activities, should graft and transform the underlying political, social and economic framework. There is also the need for identifying the best agricultural practices to boost sustainable food production, particularly among vulnerable farming communities in the country so as to prevent members of the affected communities from joining terrorist groups or community militias as a hunger coping strategy.

## CONCLUSION

Climate change and the Boko‐Haram insurgency, especially at the LCB that serves as the agricultural hub of Chad, pose challenges to food production in the country. This is further exacerbated by influx of refugees from Nigeria and Cameroon due to insecurity, adding more pressure on the already scarce resources in the country due to overcrowding; further trapping the Chadians in a vicious cycle of poverty, hunger, and insecurity. The reduction in agricultural production limits the access of displaced people and host communities to food, forcing some to adopt crisis adaptation strategies, including survival sex and joining armed groups and community militias [6], further compounding insecurity and hunger in Chad.

## AUTHOR CONTRIBUTIONS

Shuaibu Saidu Musa and Don Eliseo Lucero‐Prisno III conceived the idea. Shuaibu Saidu Musa collected and analysed the data and drafted the manuscript. Shuaibu Saidu Musa, Tchindebe Bouri Ela, Emery Manirambona, Deborah Oluwaseun Shomuyiwa, Usman Abubakar Haruna, and Abdulrahman Muhammad rotated in writing different versions of the drafts with important intellectual contributions from Don Eliseo Lucero‐Prisno III. All authors read and approved the final manuscript.

## CONFLICT OF INTEREST

Shuaibu Saidu Musa, Emery Manirambona and Deborah Oluwaseun Shomuyiwa are Youth Editorial Board members of Public Health Challenges and Don Eliseo Lucero Prisno III, the Chief Editor of the journal. They were excluded from editorial decision‐making related to the acceptance of this article for publication in the journal.
